# Protective effects of lemon and orange peels and olive oil on doxorubicin-induced myocardial damage via inhibition of oxidative stress and inflammation pathways

**DOI:** 10.3389/fphar.2025.1506673

**Published:** 2025-03-03

**Authors:** Amal I. El-Refaiy, Zainab A. Salem, Abdelnaser A. Badawy, Naief Dahran, Muhammad A. Desouky, Mohammed A. El-Magd

**Affiliations:** ^**1**^ Department of Agricultural Zoology and Nematology, Faculty of Agriculture (Girls), Al-Azhar University, Cairo, Egypt; ^2^ Nutrition and Food Science Department, Faculty of Home Economics, Al-Azhar University, Cairo, Egypt; ^3^ Department of Biochemistry, Faculty of Medicine, Northern Border University, Arar, Saudi Arabia; ^4^ Department of Basic Medical Sciences, College of Medicine, University of Jeddah, Jeddah, Saudi Arabia; ^**5**^ Department of Internal Medicine, The Brooklyn Hospital Center, Brooklyn, NY, United States; ^**6**^ Department of Emergency Medicine, Faculty of Medicine, Kafrelsheikh University, Kafrelsheikh, Egypt; ^**7**^ Department of Anatomy and Embryology, Faculty of Veterinary Medicine, Kafrelsheikh University, Kafrelsheikh, Egypt

**Keywords:** Doxorubicin, cardiomyocyte, citrus peels, olive oil, rats

## Abstract

**Background/aim:**

Compounds originating from plants, especially citrus fruits and olive oil, have anti-inflammatory, cardioprotective, and antioxidant characteristics. Doxorubicin (DOX), an anthracycline antineoplastic, induces cardiotoxicity by generating free radicals. This study aimed to evaluate the cardioprotective effects of orange (OP) and lemon (LP) peels and olive oil (OO) against DOX-induced myocardial damage in rats.

**Methods:**

Thirty adult male albino rats were randomly assigned to five groups, with six rats in each group. The control group was labeled Group I (Cnt), while Group II (DOX) got DOX intraperitoneally. Groups III, IV, and V were given a combination of DOX with OP, LP, or OO, respectively. After 28 days, cardiac biomarkers (AST, LDH, CK, cTnT), oxidative stress markers (NO, MDA), antioxidant enzyme activities (SOD, CAT, GPx), apoptotic genes (*Bax*, caspase 3, *Bcl2*), NFκB and inflammatory cytokines (TNFα, IL1β) were assessed. Histopathological analysis of the heart was also conducted.

**Results:**

DOX-treated rats showed significant functional and structural cardiac damage, characterized by elevated AST, LDH, CK, cTnT, NO, MDA, *Bax*, caspase 3, NFκB, TNFα, IL1β and reduced SOD, CAT, GPx, and *Bcl2* levels. These rats exhibited myocardial necrosis, inflammatory infiltration, mitochondrial damage, and myofibril atrophy. Treatment with OP, LP, or OO mitigated these effects, with OO providing the most substantial protection.

**Conclusion:**

These findings suggest that OP, LP, or OO can reduce DOX-induced cardiac toxicity by decreasing oxidative stress, apoptosis, and inflammation.

## Introduction

Doxorubicin (DOX), a member of the anthracycline family, is a common antineoplastic drug used to treat a variety of cancers. DOX affects cancer cells by a variety of methods, including DNA intercalation, disruption of DNA repair, production of free radicals, and their damaging effects on cellular membranes, DNA, and proteins (Vatsyayan et al., 2009). On the other hand, this effect can harm normal cells in the same manner as it damages cancer cells. The production of reactive oxygen species (ROS) is the main mechanism by which DOX induces cardiotoxicity. These ROS cause oxidative stress, inflammation, and cell death in the heart (Bruynzeel et al., 2007; Saeed et al., 2015; El-Agamy et al., 2016; Abdel-Daim et al., 2017; Shahidullah et al., 2017; Abu Gazia and El-Magd, 2018). DOX metabolism in the mitochondria results in the release of cytochrome-C, which starts apoptosis along with a disruption in the transport of mitochondrial calcium, which can induce the death of cardiomyocytes (Catanzaro et al., 2019). Thus, this drug’s clinical use was constrained by cumulative dose-dependent cardiotoxicity (Othman et al., 2021). Despite its efficacy in cancer therapy, the severe cardiac side effects limit the clinical use of DOX and necessitate the development of strategies to mitigate its toxic effects on the heart (Qiu et al., 2023).

According to recent research, there may be some benefits to using natural antioxidants in addition to chemotherapy and other chemical therapeutic agents to decrease oxidative stress damage induced by these synthetic drugs (Elmetwalli et al., 2023; Abass et al., 2024; Awad et al., 2024; Tawfic et al., 2024). Natural antioxidants have gained attention for their potential to protect against DOX-induced cardiotoxicity. Cardiovascular function can be improved with a change in lifestyle and regular consumption of healthy foods, including fruits, vegetables, and plants high in antioxidants (Topliss et al., 2002). Flavonoids like hesperidin, naringin and alkaloids like synephrine are the primary biologically active components of citrus herbs and have positive medical effects on human health (Anbazhagan et al., 2007). Citrus species exhibit antioxidant, anti-inflammatory, anti-cancer, anti-lipidemic, and antibacterial properties due to the presence of flavonoid molecules (Ekinci Akdemir et al., 2016). Researchers discovered that citrus fruit could have protective benefits for the upper respiratory tract and gastrointestinal cancer (Cirmi et al., 2018). Lemon and orange peels, rich in flavonoids and polyphenols, have shown promising effects in reducing oxidative stress and inflammation in various experimental models (González-Molina et al., 2010; Guimarães et al., 2010). Numerous useful components, particularly limonin in orange peel and pomelo peel, have been increasingly isolated and separated with the development of comprehensive citrus use (Deng et al., 2021). In addition, lemon peels contain many flavonoids that have potential health benefits (Zhang et al., 2019). Lemons are also excellent at reducing the negative effects of cancer due to their ingredients, which have strong antioxidant, antiviral, and immunostimulant properties (Tag et al., 2014).

The olive tree has been widely regarded as one species with the highest antioxidant activity through its oil, fruits, and leaves. Antioxidant compounds found in the olive tree include oleuropein, oleuropein aglycone, tyrosol, and hydroxytyrosol (Jemai et al., 2008). Olive oil, with its high content of oleic acid and polyphenols, has antioxidant, antibacterial, and anti-inflammatory properties (Millman et al., 2021) and is known to exert protective effects on the cardiovascular system (Covas et al., 2009). The risk of cardiovascular disease can be decreased by up to 10% by taking olive oil at a dose of 10 g/day (Guasch-Ferré et al., 2014). According to a clinical investigation, olive oil consumption between 10 and 50 mL per day could considerably lower blood pressure (Zamora-Zamora et al., 2018). In addition, a preclinical investigation found that a single dose of olive oil had a cardioprotective impact against cardiac remodeling and blood pressure elevation (Kamisah et al., 2015).

Based on the previously mentioned information, citrus is an economically important fruit, but the peels generated by the juice industry are a major source of agricultural waste. Their fermentation causes many economic and environmental problems, so it is worthwhile to investigate ways to use this citrus waste. Olive oil is a valuable source of antioxidants and healthy fats, and its consumption has been linked to a number of health benefits, including reduced risk of heart disease. Despite the established benefits of these natural products, their specific cardioprotective effects against DOX-induced cardiotoxicity remain underexplored. Most studies have focused on individual components rather than whole extracts, and comparative studies evaluating the effectiveness of different natural sources are limited. Therefore, this study aimed to investigate the cardioprotective potential of lemon, orange peels, and olive oil against DOX-induced myocardial damage in rats. We hypothesize that these natural products can mitigate the oxidative stress, apoptosis, and inflammation associated with DOX cardiotoxicity, providing a comparative analysis of their protective effects. The findings of this study could provide a basis for developing novel dietary strategies or adjunct therapies to reduce the cardiotoxic effects of DOX in cancer patients.

## Materials and methods

### Ethical approval

The research, which followed ARRIVE principles, was approved by the Animal Ethical Committee of the Faculty of Veterinary Medicine, Kafrelsheikh University. The committee has issued an ethical license, the VET-KFS/256/2022.

### Preparation of lemon and orange peels and olive oil

Fresh lemons, oranges, and olive oil were sourced from the local market in Tanta City, El Gharbia Governorate, Egypt. When choosing the fruits, we looked for consistent size, freshness, and the lack of mold or damage. Before being rinsed with distilled water, the fruits were given a good washing under running water from the faucet to get rid of any surface debris or pollutants. A sterile knife was used to remove the outer layers of the lemons and oranges, leaving behind just the bitter white pith. To ensure consistent drying, the peels were chopped into small, homogeneous pieces, about 1–2 cm^2^ in size. Drying the peel pieces in a hot air oven at 50°C for 72 h was done after they were spread out in a single layer on stainless steel trays. Bioactive chemicals that are sensitive to heat were preserved by choosing a low temperature. The peels were deemed to have dried to perfection when they became brittle and reached a consistent weight, meaning they did not lose any further weight as the drying process progressed. To make sure the powder was consistent in size, the dried peels were powdered using a professional electric grinder and then sieved through a 0.5 mm screen. The powder was kept in amber glass containers that were airtight at 4°C to prevent light, moisture, and oxidation until it was needed (Abdelhaliem and Sheha, 2018). Until used, the high-quality, olive oil remained in its original opaque glass container and kept at ambient temperature (25°C).

### Experimental design

Healthy male Sprague-Dawley rats (n = 30, 180 ± 5 g) housed in wire cages in a clean environment with a 12-h light-dark cycle, 70% humidity, and temperatures between 20°C and 25°C. The rats were given their food freely in designated containers to prevent dispersal. Rats were randomly assigned to five equal groups (n = 6/group). Group I (control, Cnt) was fed a basal diet for 28 days. Group II (DOX) was fed on a basal diet and intraperitoneally (i.p.) injected with 2.5 mg/kg body weight DOX (EIMC, United Pharmaceuticals, Egypt) one dose every week for 4 weeks (Abu Gazia and El-Magd, 2018). Group III (OP), group IV (LP), and group V (OO) were fed on a basal diet supplemented with dried (powder) 10% orange peels (OP), 10% lemon peels (LP), or 10% olive oil (OO) for 28 days and administrated DOX as in group II. The doses of OP, LP, and OO used in our study were selected based on findings from our pilot dose-response studies, which were conducted to identify optimal concentrations that balanced efficacy and safety. Blood samples were obtained at the end of the experiment (on day 28), and sera were separated according to the previously described methods (Alzahrani et al., 2018). Following the euthanasia of the rats through cervical dislocation while under mild ether anesthesia, their hearts were promptly dissected. The histopathological analysis required one portion to be preserved in 10% neutral buffer formalin; the second portion was snap-frozen at - 80°C for real-time PCR. In contrast, the third portion was homogenized in cold PBS, centrifuged at 5,000 g for 15 min at 4°C, and the resulting supernatant was utilized for biochemical assays.

### Biological evaluation

During the experimental period (28 days), the quantities of the diet consumed were recorded daily to calculate feed intake (FI, g). In addition, the rats’ weight was recorded weekly. At the end of the trial, feed intake and relative heart weight (heart weight (g)/final body weight (g) × 100) were determined. Weight gain (WG) and feed efficiency ratio (FER) were calculated using the following equations: WG (g) = final weight–initial weight; WG (%) = WG/initial weight × 100; and FER = WG (g)/FI (g).

### Biochemical analysis

According to what has been previously described (El-Magd et al., 2017a; Abu Gazia and El-Magd, 2018), serum levels of heart damage markers, including creatine kinase (CK), cardiac troponin (cTnT), and lactate dehydrogenase (LDH), were measured using commercially available diagnostic kits from Stanbio Laboratory Boerne, United States, and Roche Diagnostics, Mannheim, Germany, respectively. Heart homogenates were tested for levels of nitric oxide (NO), malondialdehyde (MDA), and antioxidant enzymes superoxide dismutase (SOD), catalase (CAT), and glutathione peroxidase (GPx) using kits purchased locally from Bio-diagnostic in Egypt, following methods detailed in previous studies (Abdelhadya et al., 2017; El-Magd et al., 2017a). The rat NFκB, TNFα and IL1β ELISA Kits (ab176648, ab210613, ab255730) was used to assess the level of NFκB TNFα and IL1β in cardiac homogenate according to the manufacturer’s protocol (Abcam).

### Histological and ultra-structural analysis

Ventricular samples were prepared for light microscopy by collecting and immersing them in 10% neutral-buffered formalin. Next, the specimens underwent washing, dehydration, clearing, and paraffin embedding. Using hematoxylin and eosin (H&E), 5 µm thick sections were stained. After being fixed in 2% buffered glutaraldehyde, materials were rinsed in PBS and post-fixed in 1% osmium tetroxide for transmission electron microscopy. They were encased in epoxy resin after being dehydrated in alcohol. A light microscope was used to view semithin sections that were 1 µm thick and stained with 1% toluidine blue. Using a Leica Ultramicrotome (Leica Microsystems, Vienna, Austria), ultrathin slices (50–60 nm thick) were produced, placed on copper grids, and stained with uranyl acetate and lead citrate. The grids were analyzed using a transmission electron microscope manufactured by Jeol (Jeol, Tokyo, Japan) model JEM-100.

The cardiac damage score included necrosis [score 0, no necrosis; 1, minimal necrosis (small, isolated foci); 2, mild necrosis (multiple small foci); 3, moderate necrosis (larger areas of necrosis); and 4, severe necrosis (extensive areas of necrosis)], degeneration such as vacuolization and myofibrillar loss [0, no degeneration; 1, minimal degeneration (few affected cells); 2, mild degeneration (scattered affected cells); 3, moderate degeneration (widespread affected cells); and 4, severe degeneration (extensive cellular damage)], and inflammation [0, no inflammation; 1, minimal inflammation (few inflammatory cells); 2, mild inflammation (scattered inflammatory cells); 3, moderate inflammation (focal aggregates of inflammatory cells); 4, severe inflammation (diffuse inflammatory infiltrates)] (Tan et al., 2010; Li et al., 2016). The scores for each parameter (necrosis, degeneration, and inflammation) were summed to provide a total cardiac damage score, which can range from 0 (no damage) to 12 (severe damage).

### Real-time PCR

Following the manufacturer’s procedure and a method previously reported (Abd-Allah et al., 2015), total RNA was extracted from rat cardiac tissue using the RNeasy Mini Kit (#74104) from Thermo Qiagen. Quantiscript reverse transcriptase was used to reverse transcribe 5 μg of total RNA from every sample. Gene-specific primers and the StepOnePlus real-time PCR system (Applied Biosystem, United States) were utilized to ascertain the relative expression of *Bax*, caspase3, and *Bcl2* genes from the generated cDNA (Table 1). The fold change in the expression of the target genes was determined using the reference gene, *B-actin*. Following the methodology outlined earlier (El-Magd et al., 2017b), the temperatures of the melting curves, relative expression computation, and thermal cycling conditions were all carried out. The 2^−ΔΔCT^ assay was used to find the fold change in gene expression compared to the control group for the treatment groups.

**TABLE 1:** Primer sequence of real time PCR.

Gene	Forward primer	Reverse primer
Bax	ACACCTGAGCTGACCTTG	AGC​CCA​TGA​TGG​TTC​TGA​TC
Caspase3	GGT​ATT​GAG​ACA​GAC​AGT​GG	CAT​GGG​ATC​TGT​TTC​TTT​GC
Bcl2	ATC​GCT​CTG​TGG​ATG​ACT​GAG​TAC	AGA​GAC​AGC​CAG​GAG​AAA​TCA​AAC
B-actin	AAG​TCC​CTC​ACC​CTC​CCA​AAA​G	AAG​CAA​TGC​TGT​CAC​CTT​CCC

### Statistical analysis

An analysis of variance (ANOVA) was conducted using GraphPad Prism 8 (GraphPad Software, Inc., La Jolla, CA, United States) to ascertain the significance of the groups’ differences. The assumptions of normality and homogeneity of variance were evaluated using the Shapiro-Wilk test and Levene’s test, respectively. To control Type I errors in multiple comparisons, Tukey’s Honestly Significant Difference (HSD) test was applied as a *post hoc* test. Data were shown as the mean ± the standard error of the mean (SEM), and a significance level of P < 0.05 was used to announce the results.

## Results

### Effect of treatments on growth traits

Effects of 10% lemon peels (LP), orange peels (OP), and olive oil (OO) on feed intake (FI), body weight gain (BWG), and feed efficiency ratio (FER) of rats with DOX-induced cardiotoxicity, were illustrated in Table 2. There were significant decreases (P ≤ 0.05) in FI, BWG, and FER in the DOX group compared to the control group. In contrast, the treated groups significantly (P ≤ 0.05) increased FI, BWG, and FER, with the best effect for the OO-treated group, compared to the DOX group.

**TABLE 2:** Effect of lemon (LP), orange peels (OP), and olive oil (OO) on feed intake (FI), body weight gain (BWG), and feed efficiency ratio (FER) of rats with DOX-induced cardiotoxicity.

Groups	FI (g/28 days)	BWG (g)	FER
Control	420.23 ± 10.38^a^	44.66 ± 4.72^a^	0.104 ± 0.02^a^
DOX	290.76 ± 14.04^c^	12.46 ± 1.80^c^	0.04 ± 0.02^c^
OP	386.37 ± 12.94^b^	20.44 ± 2.67^b^	0.06 ± 0.16^b^
LP	333.66 ± 29.72^b^	23.44 ± 2.22^b^	0.07 ± 0.69^b^
OO	400.66 ± 11.72^a^	30.45 ± 1.22^a^	0.07 ± 0.69^b^

The mean in the same column with completely different letters is significantly different at p < 0.05. Mean ± SEM, n = 6).

### Treatments mitigate the effects of DOX-induced cardiotoxicity indicators

The serum levels of the cardiac damage markers (LDH, CK, and cTnT) were significantly higher (P < 0.05) in rats that were injected with DOX compared to the control group, as shown in Table 3. On the other hand, the levels of these markers were significantly reduced (P < 0.05) in the treated rats relative to the DOX group. The OO group noticed the best effects, followed by the LP group. Conversely, none of the treated groups had levels comparable to the control group.

**TABLE 3:** Effect of lemon (LP), orange peels (OP), and olive oil (OO) on serum AST, LDH, CK, and cTnT of rats with doxorubicin-induced cardiotoxicity.

Groups	AST (IU/L)	LDH (U/L)	CK (U/L)	cTnT (ng/mL)
Control	84.33 ± 5.32^a^	229.61 ± 16.32^a^	184.08 ± 9.11^d^	0.09 ± 0.01^a^
DOX	315.87 ± 15.65^d^	1249.81 ± 45.19^e^	728.44 ± 27.39^a^	1.47 ± 0.11^e^
OP	209.28 ± 14.37^c^	810.83 ± 39.16^d^	410.38 ± 20.64^b^	0.67 ± 0.05^d^
LP	187.86 ± 9.23^c^	694.48 ± 32.64^c^	359.52 ± 15.71^b^	0.40 ± 0.03^c^
OO	161.57 ± 11.2^b^	461.86 ± 21.95^b^	250.27 ± 11.43 ^c^	0.28 ± 0.02^b^

The mean in the same column with completely different letters is significantly different at p < 0.05. Mean ± SEM, n = 6.

### Treatments reduce oxidative stress caused by DOX

**FIGURE 1:**
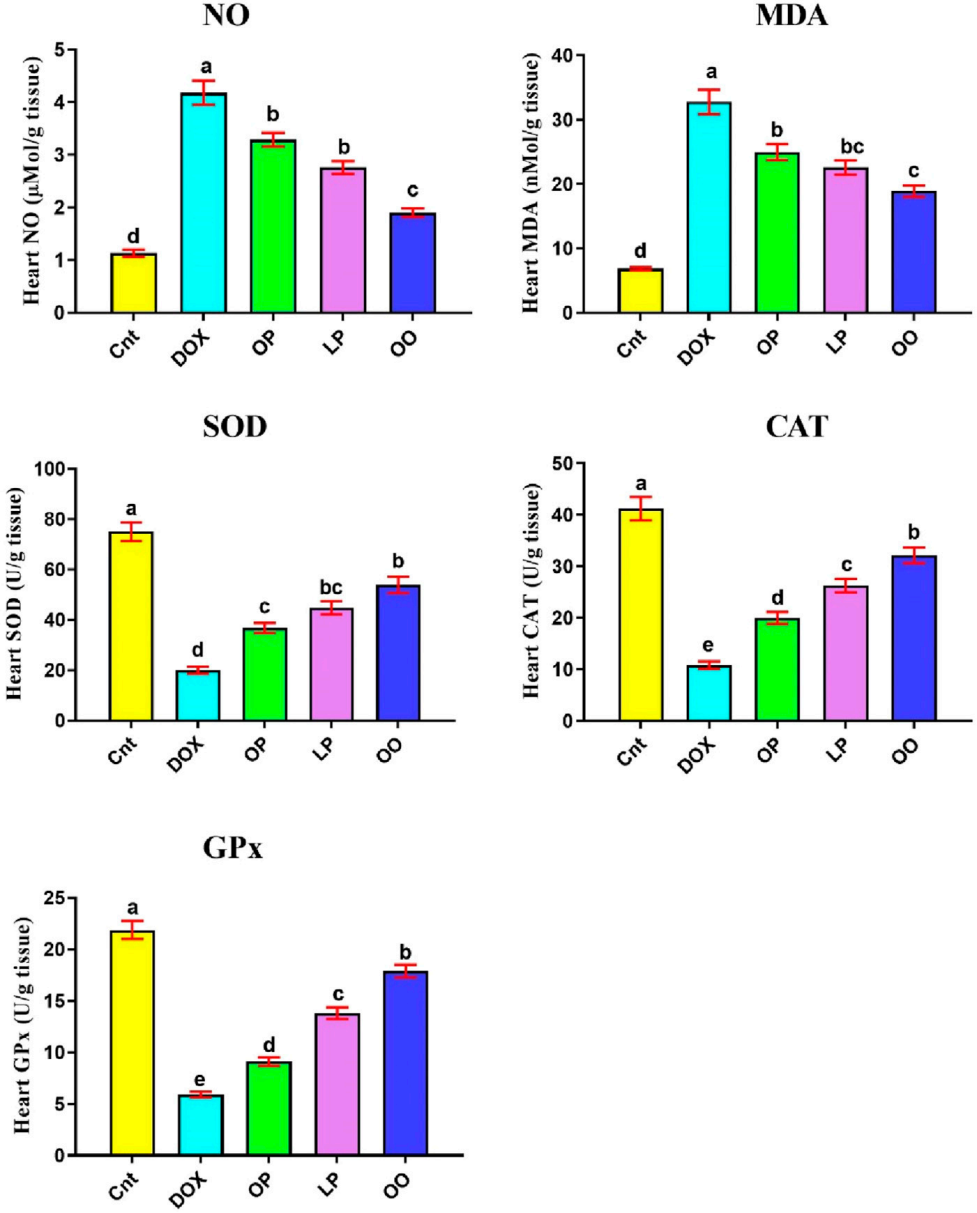
Effects of treatment with OP, LP, or OO on heart oxidant markers (NO, MDA) and antioxidant emzymes (SOD, CAT, GPx) in rats with DOX-induced cardioxicity. Data are expressed as mean ± SEM (n = 6/group). Data with different letters from “a” (representing the highest values) to “e” (representing the lowest values) are significantly different at p ≤ 0.05. All groups were compared to each other. Cnt: control group; DOX: group; OP: orane peels group; LP: lemon peels group; OO, olive oil group.

Figure 1 shows that the DOX group had significantly higher levels of oxidative stress marker (NO) and lipid peroxidation marker (MDA) in the heart than the control group. Nevertheless, compared to the control group, the DOX group had noticeably reduced levels of the antioxidant enzymes (SOD, CAT, and GPx). In contrast, compared to the DOX group, the treated groups exhibited markedly reduced levels of NO, MDA, while exhibiting noticeably elevated levels of SOD, CAT, and GPx. When comparing the three treatment groups, rats given OO had the most significant improvement, followed by those given LP and OP.

### Treatments decrease inflammation and apoptosis induced by DOX

**FIGURE 2:**
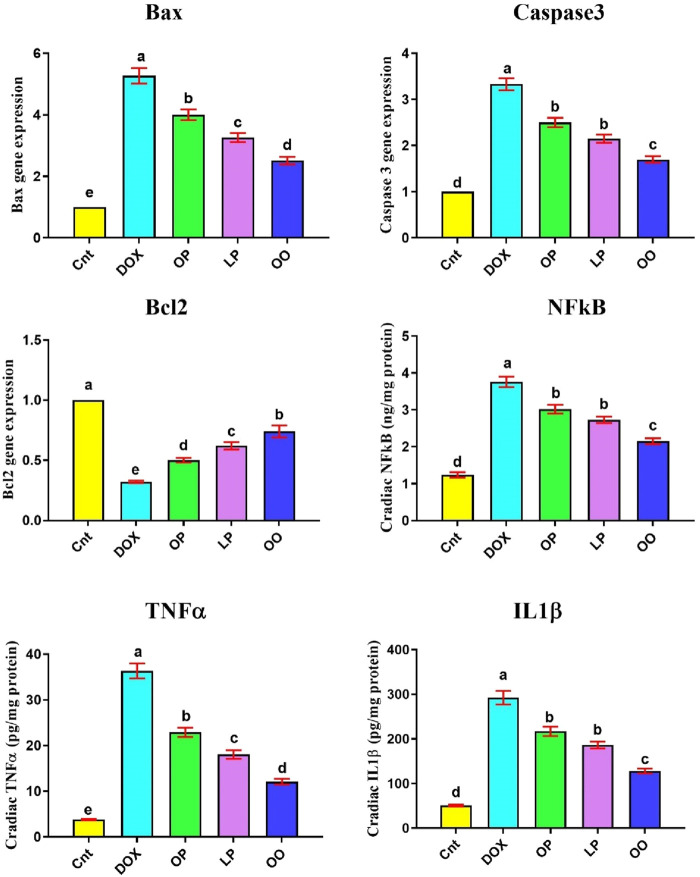
Effects of treatment with OP, LP, or OO on heart apoptotic genes (*Bax*, caspase 3, Bcl2) and inflammatory cytokines (NFκB, TNFα, IL1β) in rats with DOX-induced cardioxicity. Data are expressed as mean ± SEM (n = 6/group). Data with different letters from “a” (representing the highest values) to “e” (representing the lowest values) are significantly different at p ≤ 0.05. All groups were compared to each other. Cnt: control group; DOX: group; OP: orane peels group; LP: lemon peels group; OO, olive oil group.

Figure 2 shows that the DOX group had significantly higher levels of inflammation-related indicators (NFκB, TNFα, IL1β), and gene expression of apoptotic genes (*Bax* and caspase3) in the heart than the control group. Nevertheless, compared to the control group, the DOX group had noticeably reduced levels of the antiapoptotic gene (*Bcl2*). In contrast, compared to the DOX group, the treated groups exhibited markedly reduced levels of NFκB, TNFα, *Bax*, and caspase3, while exhibiting noticeably elevated levels of *Bcl2*. When comparing the three treatment groups, rats given OO had the most significant improvement, followed by those given LP and OP.

### Treatments repair the myocardium’s damaged histological structure caused by DOX

**FIGURE 3:**
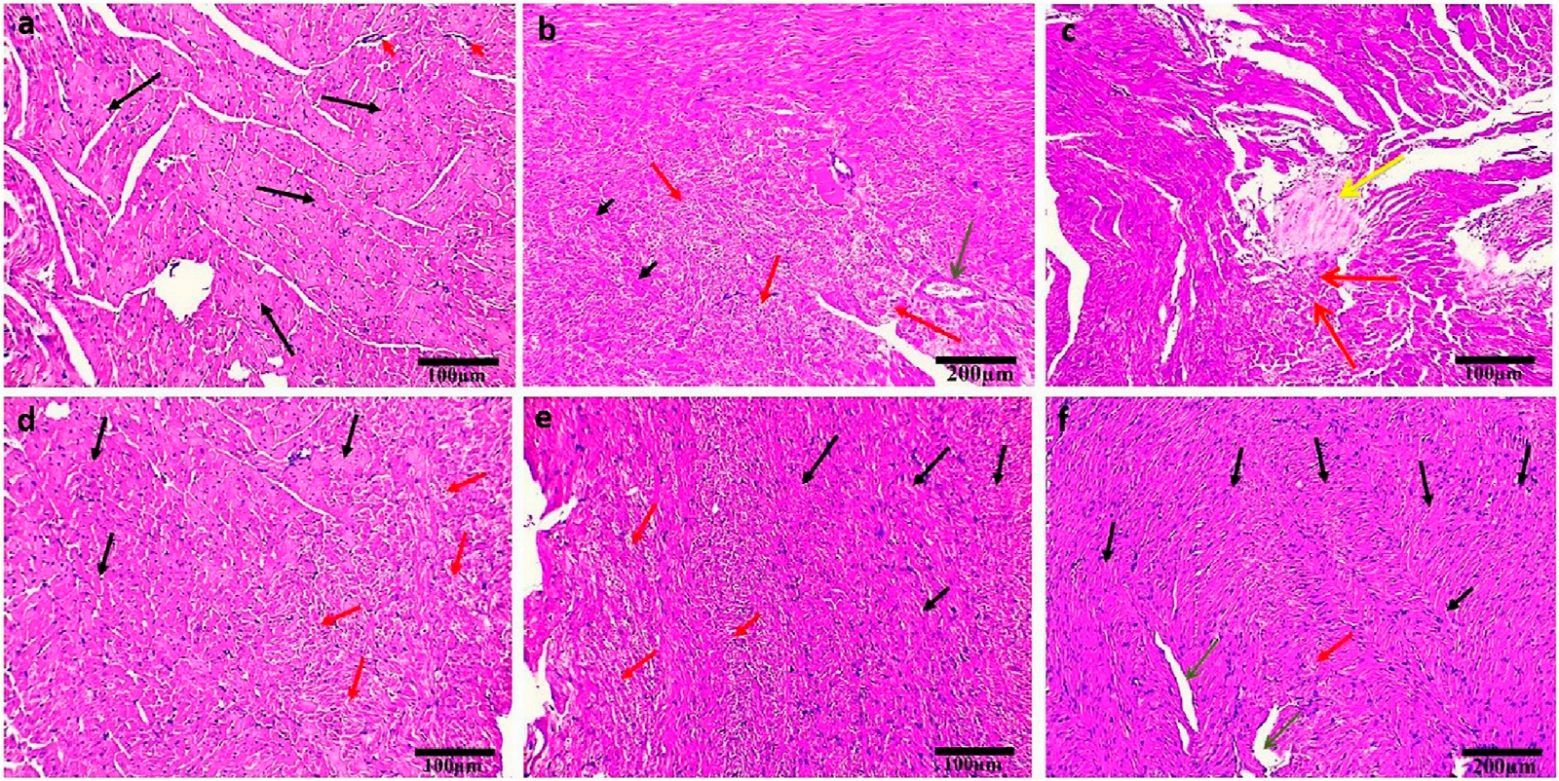
Photomicrographs of H&E-stained heart sections of control **(A)**; DOX **(B, C)**; DOX + OP **(D)**; DOX + LP **(E)**, and DOX + OO **(F)** groups. Normal myocardial bundles (black arrows), normal blood vessels (short red arrows), degeneration of myocardial cells (red arrows), areas of necrosis (short black arrows), area of hyalinization (yellow arrow), dilated blood vessels (green arrows). Scale bars = 100 and 200 μm.

The control group exhibited normal histological structure of the myocardium as revealed by a light microscopic examination of H&E-stained heart sections (Figure 3A). Cardiovascular myocytes displayed elongated vesicular nuclei in the center of acidophilic sarcoplasm (black arrows) with normal blood vessels in between myofibrils (red arrows). Sections from the DOX group revealed significant histopathological alterations in the form of wavy and irregular widely separated cardiomyocytes, severe damage of myocardial cells (red arrows), areas of necrosis (short black arrows), and hyalinization (yellow arrow), inflammatory cell infiltration in the interstitium, and dilated mild congested blood vessels (green arrow) (Figures 3B,C). On the other hand, rats treated with OP (Figure 3D) and LP (Figure 3E) showed moderate to mild degenerated myocardium (red arrows), respectively, surrounded by some normal myocardial cells (black arrows). Interestingly, rats treated with OO exhibited normal myocardial bundles (black arrows) with focal slight degeneration (red arrow) with slight dilated blood vessels (green arrows) (Figure 3F).

**FIGURE 4:**
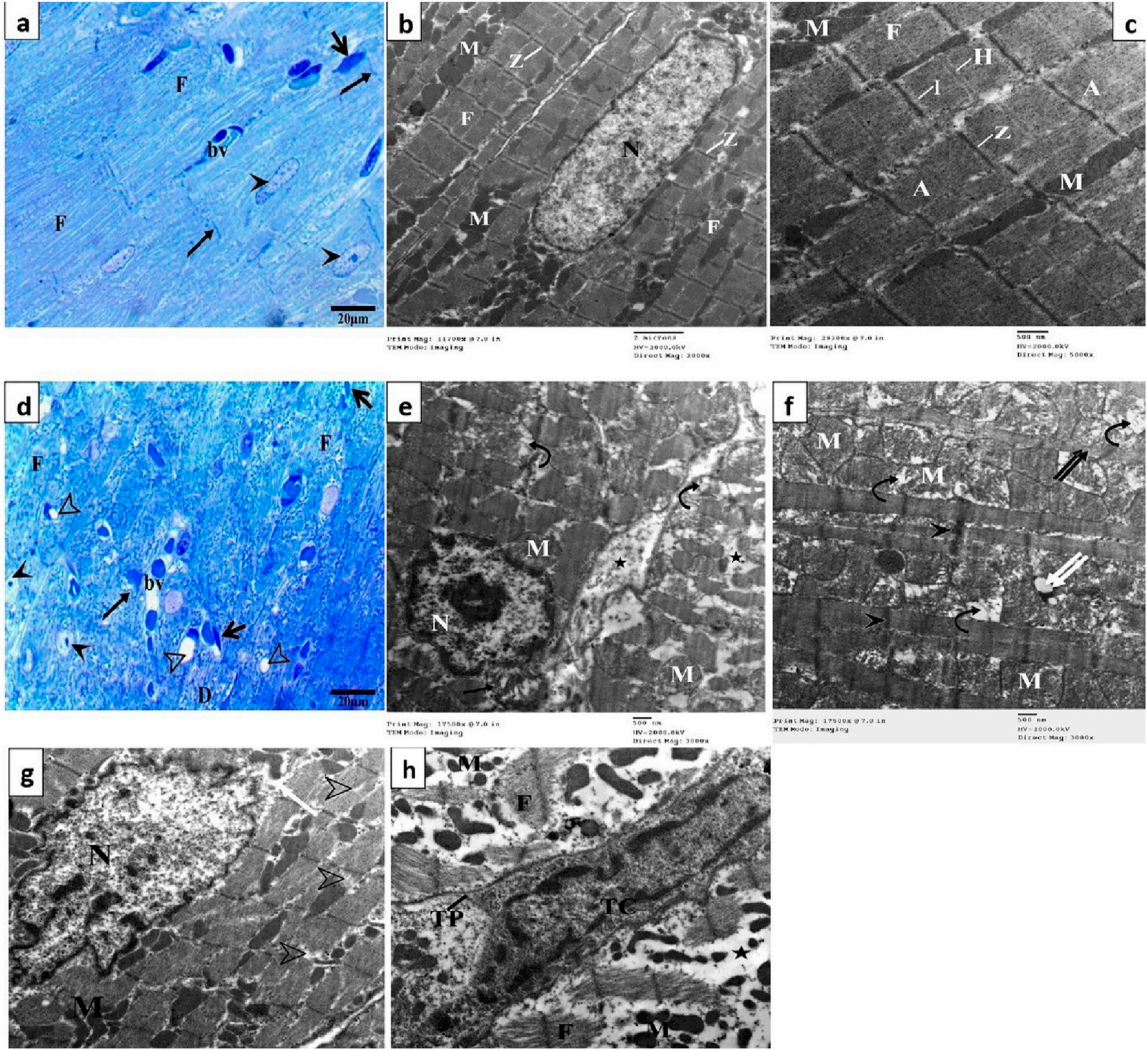
Semithin sections and electron micrographs of the control group **(A–C)** and the DOX group **(D–H)**. Semithin images showing nuclei (arrow heads), intercalated disks (black arrows), cardiomyocytes **(F)** blood capillaries (bv) and the spindle-shaped cells (short arrows) and vacuoles (hollow arrowhead). Electron micrographs showing nucleus (N) mitochondria (M), destructed cristae (curved arrows) thin myofibrils (double black arrows), thickening of Z line (arrowhead) and vacuoles (double white arrow) focal lysis of myofibrils (black arrow), perinuclear vacuolation (white arrow), nucleus of telocyte (Tc), and telopodes (Tp).

Semithin sections of the control group stained with toluidine blue revealed nearly typical morphology for cardiomyocytes (F), cross striations with central oval nuclei (arrowheads), intercalated disks (thin arrow), and delicate blood vessels (bv) with a thin wall (Figure 4A). Ultrathin sections exhibited a cardiomyocyte with a single euchromatic nucleus (N), the chromatin appeared equally distributed, and the nuclear membrane was visible. Myofibrils (F) have regular transverse striations made of dark (A) and light (I) bands divided by Z lines. There was a light H zone in the center of each A band. Between the myofibrils and in the perinuclear area, rows of many mitochondria with closely packed (M) were present (Figures 4B,C). In contrast, the DOX group showed focal disruption, disorganization of myofibril (F) with disappearance of transverse striations (D), irregular or hidden intercalated disk (arrow), vacuoles inside myocardium (hollow arrowhead), various abnormal shapes of the nuclei (arrowheads), and dilated and congested blood vessels (bv) (Figure 4D). Ultrathin sections of this group exhibited cardiomyocytes with an irregular indented nucleus (N) with abnormal condensed chromatin, swollen irregularly arranged mitochondria (M) with destructed cristae (curved arrows) and vacuoles (double white arrow), thin myofibrils (double black arrows) with thickening of Z line (black head arrow), focal lysis of myofibrils (F, hollow arrowheads), perinuclear vacuolation (white arrow), widen interstitium (*) containing nucleus of telocyte (Tc) with irregular nuclear envelope, clumps of hetrochromatin and telopodes (Tp) which had long, thin processes that encircle a region of the cytoplasm that likely contained cell debris caused by myofibril degeneration (Figures 4E–H).

**FIGURE 5:**
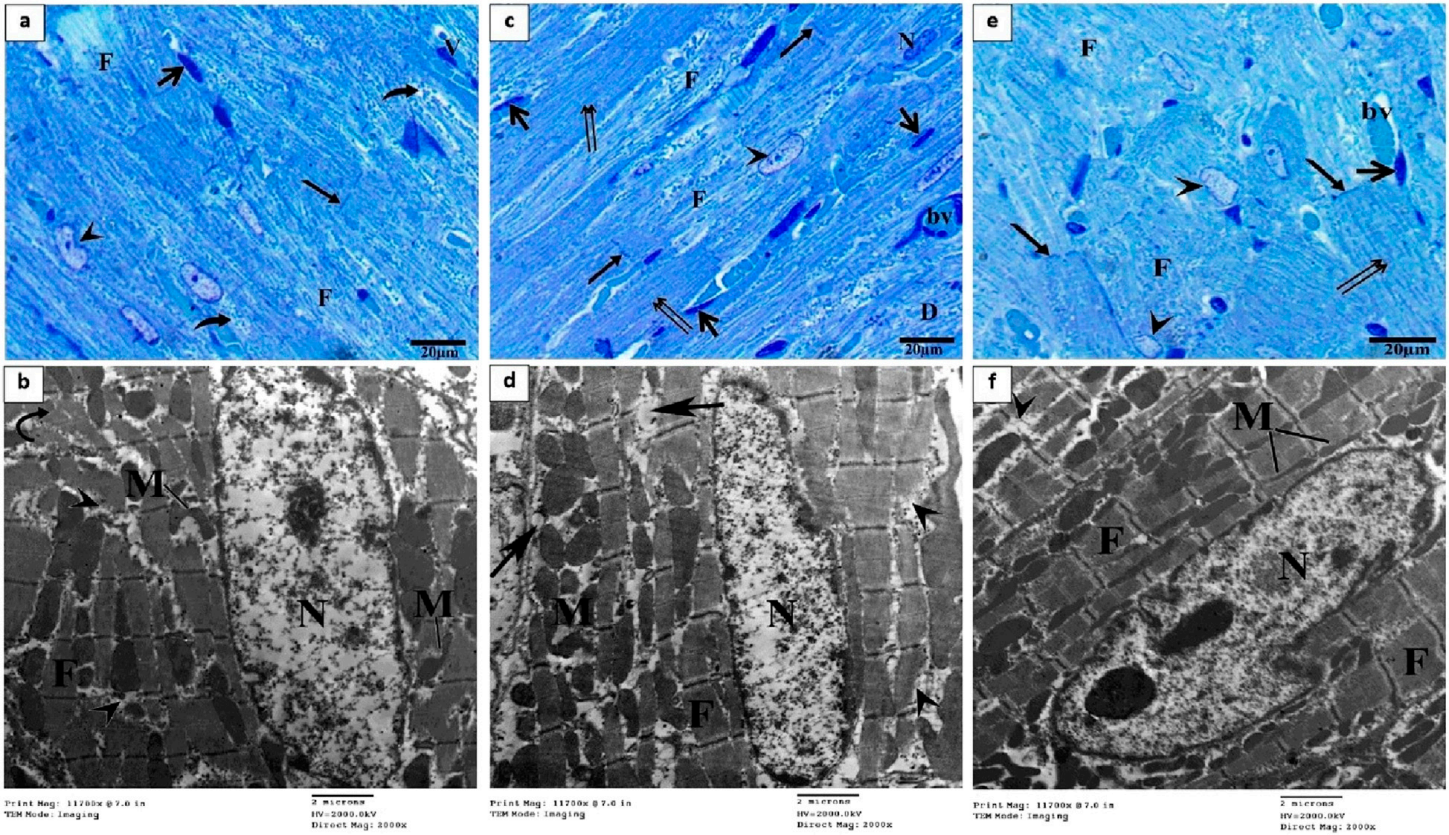
Semithin section and electron micrographs of the OP **(A, B)**, LP **(C, D)**, and OO **(E, F)** groups. Semithin images showed degenerated myocardial muscles **(F)**, distorted shaped of some nuclei (arrowhead), perinuclear vacuolation (curved arrows), normal blood vessels (bv) and intercalated disk (arrow), and normal arrangement of cardiac muscle (double arrows). Electron micrographs showing nucleus (N) mitochondria (M), degeneration of myocyte (arrowheads), vacuoles (arrows) and normal cardiomyocytes **(F)**.

Semithin section of the OP group showed large area of degenerated myocardial muscles (F) and distorted shaped of some nuclei (arrowhead), widen interstitium, perinuclear vacuolation (curved arrows), normal blood vessels (bv) and intercalated disk (arrow) (Figure 5A). The electron microscopic findings of this group showed area of myofibrils with well organized, while other loss of their striation with focal areas of degeneration (arrowheads), some mitochondria (M) lost their arrangement in some area and other had abnormal shape (Figure 5B). While LP group exhibited arrangement of some cardiac muscle (double arrows) with normal intercalated disk (arrow), other fibers (F) of cardiomyocyte were degenerated, some nuclei (N) with condensed chromatin and dilated blood vessels (bv) (Figure 5C). Most myofibrils were well organized (F) while some others were slightly degenerated (head arrows), mitochondria (M) arranged as rows with vacuoles between myofibrils and mitochondria (arrows) (Figure 5D). Section from OO group showed normal arrangement and striation of cardiac muscle (double arrows), with normal intercalated disk (arrow), dilated blood vessels (bv) and abnormal shapes of some nuclei with altered chromatin (head arrows) (Figure 5E). Electron microscopic examination revealed an increase in myofibril rearrangement and reconstruction (F), mitochondrial restructuring and arrangement within and between myofibrils (M), and oval nuclei characterized by slightly compressed chromatin and minimal nuclear membrane irregularities (Figure 5F).

**FIGURE 6:**
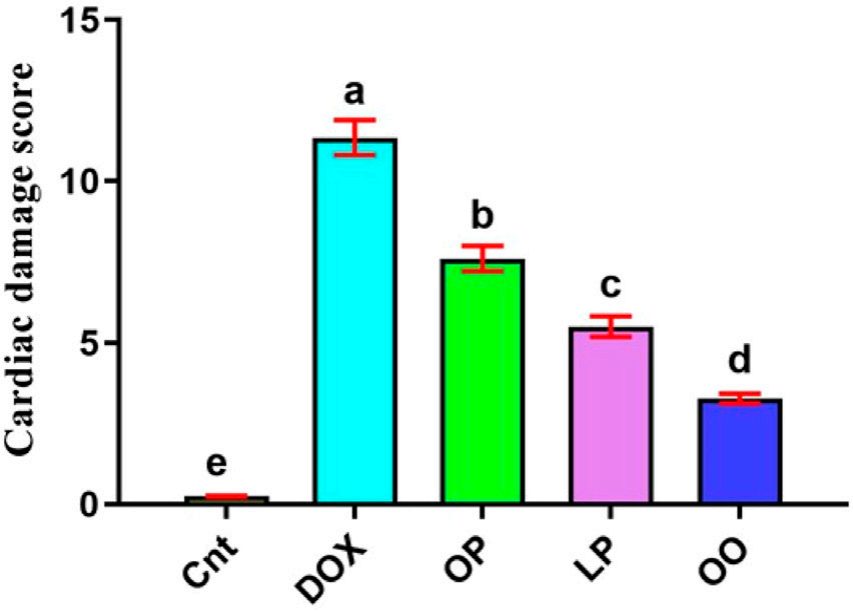
Cardiac damage score showed the effects of OP, LP, or OO in rats with DOX-induced cardiotoxicity. Data are expressed as mean ± SEM (n = 6/group). Data with different letters from “a” (representing the highest values) to “e” (representing the lowest values) are significantly different at p ≤ 0.05. All groups were compared to each other. Cnt: control group; DOX: group; OP: orane peels group; LP: lemon peels group; OO, olive oil group.

Based on the results of cardiac damage score (Figure 6), DOX-intoxicated rats exhibited significantly (P < 0.05) higher damage score than the control group. In contrast, rats treated with LP, OP, or OO showed significantly (P < 0.05) reduced damage score, with lowest score in OO group followed by LP group, compared to the DOX group.

## Discussion

DOX, an anticancer drug, has been reported to increase oxidative stress, which can affect different organs, including the heart. Here, we investigated the toxic effects of DOX on heart function and structure in rats and evaluated the potential protective effects of lemon and orange peels and olive oil. We found that lemon and orange peels and olive oil can significantly mitigate DOX-induced cardiotoxicity in rats as revealed by reduced cardiac biomarkers (AST, LDH, CK, cTnT), oxidative stress markers (NO, MDA), apoptotic markers (*Bax* and caspase 3), and inflammatory cytokines NFκB, TNFα, IL1β while enhancing antioxidant enzyme activities (SOD, CAT, GPx) compared to the DOX group.

The present results indicate that the DOX group showed a significant decrease in FI compared to other groups, consistent with previous research (Arafa et al., 2014). The olive oil group had FI values closest to the control group. DOX treatment resulted in a significant decrease in BWG, while rats fed with orange or lemon peels showed a significant increase in BWG. The use of DOX simultaneously with the orange peel extract maintained the body weight of the nude mice, which illustrates the protective effects of the orange peel extract (Tajaldini et al., 2020). DOX also caused a significant reduction in FER, aligning with Ghibu et al. (2012), who reported that cumulative DOX doses led to reduced body weight and food consumption due to induced anorexia. Moreover, we observed that DOX significantly elevated serum levels of cardiac damage markers (LDH, AST, CK, and cTnT) compared to the control group, consistent with findings of other studies (Abu Gazia and El-Magd, 2018; Xing et al., 2022). This increase is attributed to the oxidative stress and free radical release caused by DOX, which compromise cardiac cell membranes, leading to enzyme leakage into the bloodstream. Conversely, these markers were significantly reduced in all treatment groups, with the most notable improvement observed in the olive oil group. Similarly, Hammad et al. (2018) reported that active components in orange peels decreased serum AST, cTnT, and LDH levels, while Abdelhaliem and Sheha (2018) demonstrated similar effects with lemon peel. Generally, a drop in these biomarker levels indicates that the treatments have improved heart health.

Our results revealed potent DOX cardiotoxic effects in rats as evidenced by increased levels of oxidative stress (high levels of NO and MDA and low activity of SOD, CAT, and GPx). When cytochrome P450 reductase and NADH dehydrogenase convert DOX’s quinine moiety to its corresponding semiquinone form, it can trigger ROS production (Kalishina et al., 2003). The DOX semiquinone form is converted to quinine by molecular oxygen oxidation, which in turn produces superoxide radicals. Consistent with our results, Abu Gazia and El-Magd (2018), Ayaz et al. (2004), and El-Agamy et al. (2016) have also demonstrated that DOX treatment causes a substantial rise in cardiac MDA levels. Elevated MDA causes lipid peroxidation, which in turn destroys the cell membranes of cardiomyocytes and releases LDH, CK, and cTnT into the bloodstream because phospholipids are the primary components of cell membranes. By activating endogenous antioxidant enzymes, the body counteracts excessive ROS. However, DOX also targeted this defense mechanism, and a decline in these enzymes suggests that cardiomyocytes suffer oxidative damage because cells can’t remove free radicals (Tokarska-Schlattner et al., 2006). In agreement with our findings, after DOX induction, NO production was significantly increased (Pinto et al., 2007). In contrast, antioxidant enzyme activities were increased in response to the administration of olive oil, lemon, and orange peels, while NO and MDA levels were decreased. This effect can be attributed to the presence of potent antioxidant compounds in olive oil, such as oleuropein and hydroxytyrosol (Utarı et al., 2022), and in citrus peels, such as quercetin, hesperidin, L-carnitine, and silybin (Aziz, 2021; Abd El-Motelp, 2022). Olive oil demonstrates a cardioprotective effect against DOX-induced cardiotoxicity (Almalki et al., 2020), primarily due to its rich content of antioxidant compounds, such as oleuropein and hydroxytyrosol (Rus et al., 2017). Rather than diminishing DOX’s anticancer efficacy, the antioxidants in olive oil have been found to enhance its therapeutic effects, making it a potential synergistic adjunct in chemotherapy (El-Kammar and Ghazy, 2018). Oleic acid has been shown to increase plasma HDL levels while reducing LDL levels (Jimenez-Lopez et al., 2020). Our findings suggest that with their higher L-carnitine content, lemon peels exhibited more pronounced benefits than orange peels. Hesperidin, concentrated in the white pith of citrus fruits, acts as an antioxidant and anti-inflammatory agent and has been associated with reduced risks of heart disease and cancer (Khamis et al., 2018; Selim et al., 2019; Badawy et al., 2021; Sajadi et al., 2021). Therefore, whole citrus peels were used in this study to maximize these beneficial effects. Citrus peels and seeds are abundant in phenolic compounds, with the peels containing higher levels of flavonoids than the seeds (Sawalha et al., 2009). Numerous phytochemicals in orange peel extract are recognized for their antioxidant properties and ability to protect against oxidative stress (Bae and Kim, 2016). These natural antioxidants have protective properties against DOX-induced cardiac damage (Hammad et al., 2018; Patel et al., 2018).

Analyses using light and electron microscopy showed that DOX severely damaged the myocardium by compromising its structural integrity. In the group that received DOX, light microscopy revealed cardiomyocytes that were wavy, irregular, and widely separated; there was also focal necrosis with areas of hyalinization, tissue fibrosis, inflammatory cell infiltration, and clogged blood vessels. This finding aligns with prior research (Abu Gazia and El-Magd, 2018; Qin et al., 2022; Utarı et al., 2022). According to our results and those of Safdar et al. (2017) and Babaei et al. (2020), TEM imaging further supported these findings, showing broken cristae, enlarged mitochondria, and irregularly shaped nuclei in disturbed cardiomyocytes. Because they produce most of the superoxide that the heart cells release when exposed to DOX, the mitochondria in myocardial cells are the first organelles to suffer oxidative damage, which in turn causes mitochondrial malfunction and cell death (Utarı et al., 2022). Our results of the molecular study showed that the cardiac tissue underwent mitochondrial-dependent apoptosis, as shown by the upregulation of *Bax* and caspase three and the downregulation of *Bcl2*. Rats treated with orange peel exhibited mild myocardial alterations, such as abnormal mitochondria and loss of striation. In contrast, those treated with lemon peel showed minimal alterations, effectively preventing DOX-induced cardiac injury. Treatment with olive oil provided the greatest cardioprotection, as evidenced by only minor histopathological changes, and lowest expression of *Bax* and caspase three genes, and highest expression of the *Bcl2* gene. This protective effect is attributed to the high oleuropein content in olives and olive oil (Hamza et al., 2021). Studies have shown that oleuropein significantly reduces structural changes in cardiomyocytes, supporting its role as a potent antioxidant that mitigates DOX toxicity by decreasing *iNOS*, *Bax* expression, and NO production (Andreadou et al., 2007).

The protective mechanisms of these natural products against DOX-induced cardiotoxicity also include the suppression of inflammatory pathways (lower levels of NFκB, TNFα, and IL1β). This aligns with previous studies suggesting that antioxidants can counteract DOX-induced free radical formation, apoptosis, and inflammation in cardiac tissue (Andreadou et al., 2007; Guimarães et al., 2010; Abu and El-Magd, 2018; Hamza et al., 2021). The observed cardioprotective effects can be attributed to the high content of bioactive compounds in these natural sources, including flavonoids, polyphenols, and oleic acid, known for their antioxidant and anti-inflammatory properties (Covas et al., 2009; González-Molina et al., 2010; Almalki et al., 2020)**.** The histopathological examination corroborated these biochemical findings, showing that lemon and orange peels and olive oil preserved myocardial architecture, reduced inflammatory cell infiltration, and minimized mitochondrial damage and myofibril lysis. Among the treatments, olive oil exhibited the most pronounced protective effects, possibly due to its rich composition of phenolic compounds and unsaturated fatty acids, which have been shown to modulate oxidative stress and inflammation more effectively than other natural sources (Covas et al., 2009). Thus, it would be recommended to add olive oil to salads and vegetables and eat whole citrus fruit with its peel to improve heart health.

Oxidative stress induced by DOX could activate NFκB, which subsequently regulates TNFα, IL1β and pro-apoptotic genes *Bax* and caspase 3 (Gloire et al., 2006; Tan et al., 2010). Similarly, the inhibition of PI3K/AKT signaling may suppress anti-apoptotic proteins such as Bcl2 (Franke et al., 2003). The observed reduction in Bax and caspase three and the elevation of Bcl2 levels following treatment with citrus peels and olive oil suggest a potential modulation of these pathways. Notably, the high antioxidant and anti-inflammatory properties of these treatments could mediate upstream effects by reducing ROS production and suppressing NF-κB activation.

Several limitations should be considered when interpreting these results. First, the study was conducted on a small sample size of rats, which may limit the generalizability of the findings. Second, the study did not investigate the long-term effects of these natural products beyond the 28-day experimental period. Third, the exact molecular mechanisms underlying the cardioprotective effects were not explored in depth. Fourth, our study did not explore whether these natural treatments interfere with DOX’s anticancer effects. Fifth, this study also lacks comprehensive dose-response studies that could further elucidate the therapeutic potential of lemon and orange peels and olive oil and their mechanisms of action. Future research should include a larger sample size, extended observation periods, and molecular studies to better understand the pathways involved in the protective effects of these natural products. Future studies could also investigate whether these cardioprotective treatments reduce DOX’s efficacy against tumors, balancing cardioprotection with cancer treatment.

## Conclusions

This study highlights the potential of lemon and orange peels and olive oil in protecting against DOX-induced cardiotoxicity by reducing oxidative stress, apoptosis, and inflammation in cardiac tissue. Among the tested treatments, olive oil showed the most significant cardioprotective effects. These findings suggest that incorporating these natural products into the diet could be a beneficial strategy for mitigating the cardiotoxic effects of DOX in cancer patients. Further research is warranted to explore their potential use as complementary therapies in clinical settings.

## Data Availability

The original contributions presented in the study are included in the article/supplementary material, further inquiries can be directed to the corresponding author/s.

## Abbreviations

AST, Aspartate aminotransferase; Bax, Bcl2-associated X; Bcl2, B-cell leukemia/lymphoma 2 protein; CAT, Catalase; CK, Creatine kinase; cNtN, Cardiac troponin; DOX, Doxorubicin; GPx, Glutathione peroxidase; IL1β, Interleukin 1 beta; LDH, Lactate dehydrogenase; LP, Lemon peels; NO, Nitric oxide; NFκB, Nuclear factor kappa-light-chain-enhancer of activated B cells; MDA, Malondialdehyde; OO, Olive oil; OP, Orange peels; ROS, Reactive oxygen species; SOD, Superoxide dismutase; TNFα, Tumor necrosis factor-alpha.
